# Circular RNA circ_0000020 promotes osteogenic differentiation to reduce osteoporosis via sponging microRNA miR-142-5p to up-regulate Bone Morphogenetic Protein BMP2

**DOI:** 10.1080/21655979.2021.1949514

**Published:** 2021-07-16

**Authors:** Rongkui Zhou, Shichang Miao, Jun Xu, Liping Sun, Yaofei Chen

**Affiliations:** Department of Orthopedic, Jiangyin Hospital Affiliated to Nanjing University of Chinese Medicine, Jiangyin City, Jiangsu Province, China

**Keywords:** Circ_0000020, miR-142-5p, osteoporosis, bone morphogenetic protein bmp2, osteogenic differentiation, bone marrow-derived mesenchymal stem cells

## Abstract

This study was designed to study functions of Circ_0000020 during osteogenic differentiation. First, we used RT-qPCR to detect the expression of Circ_0000020, miR-142-5p and osteogenesis-related genes, whereas western blot analysis detected the expression of osteogenesis markers after the osteogenic differentiation of primary BMSCs isolated from rats. Alkaline phosphatase (ALP) activity and alizarin red Sstaining validated osteoblast phenotypes. Flow cytometry was used to detect cell apoptosis. Sh-Circ_0000020 was used to study the function of Circ_0000020 in osteogenic differentiation of BMSCs. Luciferase assay and RNA immunoprecipitation were used to validate the interaction between Circ_0000020 and miR-142-5p, and BMP2 and miR-142-5p. Co-transfection of miR-142-5p and sh-Circ_0000020 was used to verify the downstream signaling pathway. Circ_0000020 expression was up-regulated during osteogenic differentiation, whereas miR-142-5p expression was significantly decreased. Silencing Circ_0000020 inhibited osteogenic differentiation and promoted apoptosis, and inhibited ALP activity and mineralization ability. Moreover, Circ_0000020 interacts directly with miR-142-5p which binds to the BMP2 3ʹUTR and inhibits its expression. Additionally, co-transfection of miR-142-5p inhibitors and sh-Circ_0000020 rescued down-regulated BMP2, increased the expression osteogenesis-related gene expressions, and thereby rescued the inhibition of osteogenic differentiation induced by Circ_0000020 silencing. Furthermore, co-transfection of miR-142-5p inhibitors and sh-Circ_0000020 reversed Circ_0000020 silencing-induced downregulation of p-Smad1/5/8, Runx2, and Osterix protein levels. Circ_0000020 regulates BMP2 expression through sponging miR-142-5p as ceRNA, thereby positively regulating BMSCs osteogenic differentiation through Circ_0000020/miR-142-5p/BMP2/SMAD-dependent signaling pathway.

## Graphical abstract

**Figure:**
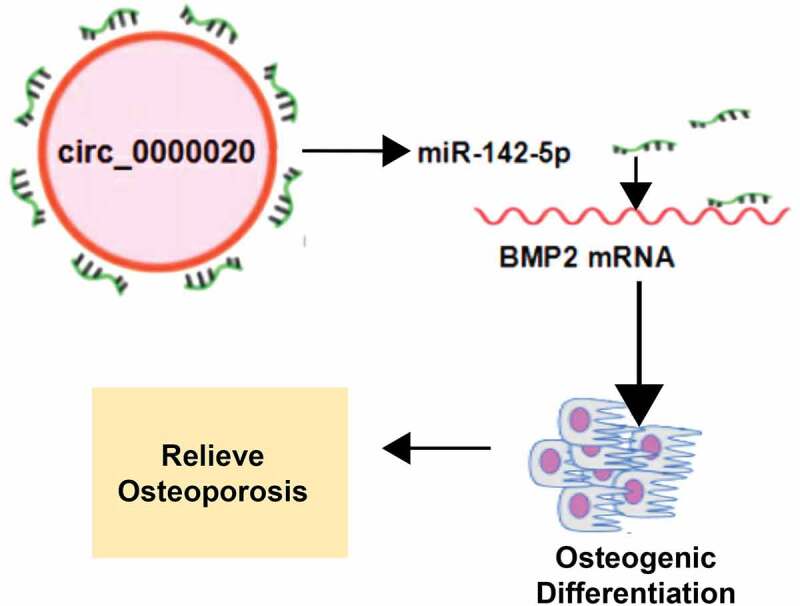

## 1 Introduction

Osteoporosis (OP) is a systemic metabolic bone disease which is characterized by the decreased bone mass and toughness, destructed bone microstructures, and increased bone fragility [1]. With the increased population aging, OP patients are continuously increasing since last few decades. It is estimated that >25 million Americans are suffering from OP, which results in approximately 1.5 million fractures annually. Approximately 70% of people with OP are women [2]. OP mainly affects middle-aged and elderly people by causing serious health problems, and brings a heavy burden to families and society [3]. Bone marrow-derived mesenchymal stem cells (BMSCs) are considered as the main source of osteoblastic progenitor cells which take part in bone repairing process. BMSCs have strong proliferation ability, and multidirectional differentiation potential with broad application prospects [4]. Therefore, promoting BMSCs differentiation is one of the vital treatments strategies to alleviate OP.

Circular RNAs (circRNAs), closed-loop structure formed by reverse splicing and covalent bonding, are RNA [5] which perform complex biological functions. CircRNAs participate in various cellular processes, including cell proliferation, EMT, and development, and regulate osteogenic differentiation [6,7]. For example, Hsa_circ_0006859 upregulates ROCK1 via sponging miR-431-5p to inhibit osteogenesis, and promotes fat formation [8]. Additionally, hsa_circ_0026827 promotes Dental Pulp Stem Cells (DPSCs) osteoblast differentiation through Beclin1 and RUNX1 signaling pathway by sponging miR-188-3p, which implied a new therapy for OP [9].

MicroRNAs (miRNAs; about 22nt), non-coding small RNAs, regulate gene expression of their target mRNAs by complementary base pairing with three prime untranslated region (3ʹUTR) of target mRNAs, thus participate in various biological processes, including differentiation and development, metabolism, proliferation and apoptotic cell death [10,11]. Based on previous studies, miRNAs are closely associated with OP [12,13]. Additionally, several studies have found that miR-142-5p inhibits osteoblast differentiation and promotes OP progression [14]. Based on previous studies, LncMIR99AHG is up-regulated in OP with a binding site with miR-142-5p [15]. This indicates that miR-142-5p is regulated by upstream non-coding RNA, and therefore plays an inhibitory role in OP.

It has been reported that circRNAs have rich miRNA-binding sites; therefore, they have ability to play the role as competing endogenous RNA (CeRNA) by acting as miRNA sponges in cells, and regulate the pathogenesis of several diseases [16,17]. This ceRNA networking eliminates the regulatory and inhibitory effects of the miRNAs on their target genes and thus increasing the expression level of the target genes. It is widely accepted that CircRNA-miRNA network has a close relationship with osteogenic differentiation [18]. In a recent study, it was reported that Circ_0000020 promotes glioma cell growth, and links with poor prognosis [19]. However, the role of Circ_0000020 in regulating osteogenic differentiation is not studied well. This study determined the Circ_0000020-dependent molecular mechanism involved in regulating BMSCs osteogenic differentiation. Briefly, in this study, we tried to uncover the potential roles of Circ_0000020 during osteogenic differentiation of BMSCs, and its potential role in ceRNA network by binding with miR-142-5p which directly binds to the 3ʹUTR of Bone Morphogenetic Protein BMP2 (BMP2). Furthermore, we also uncovered the potential downstream signaling pathway. This is the first report to describe the regulatory role of Circ_0000020/miR-142-5p/BMP2 axis in BMSCs osteogenic differentiation, thus providing new theoretical basis for developing new drug targets.

## 2 Materials and Methods

### 2.1 BMSCs isolation, culture, and identification

Twenty clean-grade healthy female Sprague Dawley (SD) rats, 3 months old (200–220 g), were obtained from the Experimental Animal Center of Guangzhou University of Chinese Medicine. Later, animals were immersed in 70% ethanol for 15 minutes to separate femur and tibia under aseptic conditions, as well as to remove the soft tissue. Both ends of femur and tibia were resected to wash out the marrow with L-modified Eagle medium. The marrow collected from 4 to 5 femurs and tibias was placed into the same beaker to mix thoroughly and transferred to centrifuge tubes for 10 minute-centrifugation at 300 g. Later the supernatant was removed to resuspend in D-Hanks, and centrifuged again to remove the remaining supernatant and added 4 mL of D-Hanks to mix evenly. Afterward, 4 mL of Percoll separation solution mixed at 0.56:0.44 was dispensed into 10 mL centrifuge tubes to centrifuge at 900 g for 30 minutes, and collected mononuclear cell layer, which was later washed with Dulbecco’s Modified Eagle Medium (DMEM) containing 10% Fetal Bovine Serum (FBS) twice, and inoculated statically into 25 mL culture flask (4 × 10^5^/cm^2^) to cultivate in incubators at 37°C with 5% CO_2_ with saturated humidity. The culture medium was L-DMEM containing 10% FBS, replaced for the first time after 3 days to remove suspended cells. The medium was changed once every 2 to 3 days. After the 70% to 80% confluency, cells were digested with 0.25% trypsin at room temperature for 2 to 3 minutes, and further passaged at 1 × 10^4/^cm^2^ to the third generation, and 1 × 10^7^/ cm^2^ cells were obtained [20].

### 2.2 BMSCs induced osteogenesis

After being cultured in DMEM supplemented with 10% FBS, the cells were placed in incubators with 50 U/mL penicillin and 100 μg/mL streptomycin at 37°C with 5% CO_2_. Once BMSCs reached 70% ~ 80% confluency, 50 μg/mL vitamin C + 10 mmol/L β-glycerophosphate sodium+0.1 μmol/L dexamethasone were used to induced osteogenesis, and the induction medium was changed every 3 days [21].

### 2.3 Cell transfection

To suppress Circ_0000020 expression, short hairpin(sh)-RNA targeting Circ_0000020 (sh Circ_0000020) sequence was constructed in U6/GFP/Neo plasmids, plasmids carrying non-targeting sequences were transfected and served as a control (sh-NC). Sh-Circ_0000020, miR-142-5p inhibitor and its NC were purchased from GenePharma. In short, BMSCs were inoculated in 2 mL of osteogenic induction medium, and sh-Circ_0000020 or miR-142-5p inhibitors were added to the cells with 90% confluency via lipofectamine 2000 (Invitrogen). After 4 hours of transfection at 37°C with 5% CO_2_, the previous medium was replaced with fresh media and cultured for 48 hours [20].

### 2.4 Real-time quantitative polymerase chain reaction (RT-qPCR)

Total RNA was extracted with Trizol reagent (Invitrogen), DNA was removed via TurboDNase kit (Ambion), and the extracted RNA was quantified with NanoDrop. PrimeScript real-time (RT) kit (Takara Bio) was used to synthesize complementary DNA from 1000 ng of total RNA. RT-qPCR was performed on ABI 7900 system by using SYBR Select Master Mix (Applied Biosystems, U.S.) GAPDH was used as the standardization internal control for Circ_0000020 and BMP2 mRNA, while U6 was used as internal control for miR-142-5p. CT value was calculated in reference to 2− ΔΔCt [21]. The primer sequences used in this study are listed in Table 1.

**Table 1.** List of primers used in this study

Gene name	Forward Primer	Reverse Primer
Circ_0000020	5’-GAGAGGATGTACGGCCAGAG-3’	5’-AAACTTTCCGGAGCCTCTTC-3’
miR-142-5p	5’-GGATCATAAAGTAGAAAA-3’	5’-CAGTGTGTCGTGGAGT-3’
BMP2	5’-GCAAAGAAAAGGAACGGACATT-3’	5’-GGGAAGCAGCAACGCTAGAA-3’
OPN	5’-GCCGAGGTGATAGTGTGGTT-3’	5’-AACGGGGATGGCCTTGTATG-3’
OCN	5’-ACACCATGAGGACCATCTTTC-3’	5’-CGGAGTCTGTTCACTACCTTATT-3’
RUNX2	5’-AAGTGCGGTGCAAACTTTCT-3’	5’-ATGACTCTGTTGGTCTCGGTG-3’
U6	5’-CTCGCTTCGGCAGCACA-3’	5’-AACGCTTCACGAATTTGCGT-3’;
GAPDH	5’-GAAGGTGAAGGTCGGAGTC-3’	5’-GAAGATGGTGATGGGATTTC-3’

### 2.5 Alkaline phosphatase (ALP) staining and activity detection

BMSCs were subjected to osteogenic differentiation by culturing for 7 days. Differentiation-induced osteoblasts were seeded in 6-well plates, and subjected to ALP activity determination in reference to the instructions of ALP detection kit (Abcam). Later, cells were washed with phosphate-buffered saline (PBS) to stain with hematoxylin. After washing and air-dry, BMSCs were observed under light microscopes [22].

### 2.6 Alizarin red S staining and mineralization accumulation detection

After 7 d and 14 d of culturing BMSCs, cells were washed twice with PBS, fixed with 10% formaldehyde at room temperature for 15 minutes, washed twice with double-distilled water, and finally 40 mM alizarin red S solution was added (1 mL/well) for 20-min incubation at room temperature with slight shaking. Then, the dye that was not completely bound was removed, and rinsed with double distilled water to shake for 5 min (4 times). The excess double distilled water was removed, and inverted microscopes were used to observe the cells [22].

### 2.7 Cell apoptosis

To determine the cell apoptosis rate, AV/7-AADA proptosis detection Kit (Southern Biotechnology, U.S.) was used according to the manufacturer’s instructions. The treated cells were digested and centrifuge, washed twice with cold PBS, and re-suspended to 1 × 10^6^ cells/mL using binding buffer. Later, double staining was performed using AV/7-AAD, and incubated in the dark at room temperature for 15 minutes. Finally, flow cytometry (BD Biosciences) was applied to analyze the stained cells via Fluorescence-activated cell sorting (FACS) Calibur Flow Cytometer (BD Biosciences) [23].

### 2.8 Western blot assay

Radioimmunoprecipitation assay buffer (RIPA; Beyotime) buffer was used to extract total cell protein. Later, protein was separated through electrophoresis and transferred to polyvinylidene difluoride (PVDF) membrane (Millipore). After blocking with 5% skim milk for 2 h, the membranes were incubated with the primary antibodies, that is, Anti-Smad 1/5/8 (ab126761, 1:1000), Anti-Runx2 (ab192256, 1:1000), Anti-Osterix (ab22552, 1:1000) and anti-GAPDH (ab8245, 1:2000) for incubation overnight at 4°C. The next day, the membranes were incubated with the corresponding secondary antibodies at room temperature for 2 hours, and the immunoreactive bands were exposed via enhanced ECL (Thermo Fisher Science). Image J software (NIH, Bethesda) was used for analysis [22].

### 2.9 Dual-luciferase reporter assay

After amplification, Circ_0000020 and BMP2 sequences were cloned into pmirGLO plasmids (Promega) to obtain pMIR-Circ_0000020-wt and pMIR-BMP2-wt. To obtain the mutant plasmids (pMIR-Circ_0000020-mut), the fragments containing mutant target regions were designed and cloned into pmirGLO to obtain pMIR-Circ_0000020-mut and pMIR-BMP2-mut. Later, MiR-142-5p mimics or miR-NC were co-transfected with pMIR-Circ_0000020-wt or pMIR-BMP2-wt, or their respective mut plasmids into HEK-293 T cells using Lipofectamine® 2000. The relevant luciferase activity was analyzed using Dual-Luciferase Reporter Assay System (Promega) 48 h after transfection [24].

### 2.10 Ago RNA Immunoprecipitation

Magna RIP RNA binding protein immunoprecipitation kit (Millipore) was used to determine the interaction between Circ_0000020 and miR-142-5p. Antibodies used for RIP detection were anti-AGO2 antibody and NC IgG (Millipore). The co-precipitated RNA was subjected to the cDNA synthesis, and the amplification and quantification was achieved using RT-qPCR [25].

### 2.11 Statistical analysis

Statistical analysis was performed via GraphPad Prism 8 (Version X). All experiments were performed in triplicate. This work applied student’s t test to analyze intergroup differences, one-way analysis of variance (ANOVA) for comparison among multiple groups, followed by post-group test (least significant difference). *P* < 0.05 was considered statistically significant [26].

## 3 Results

### 3.1 Expression of Circ_0000020 is increased during BMSCs osteogenic differentiation

**Figure 1.**
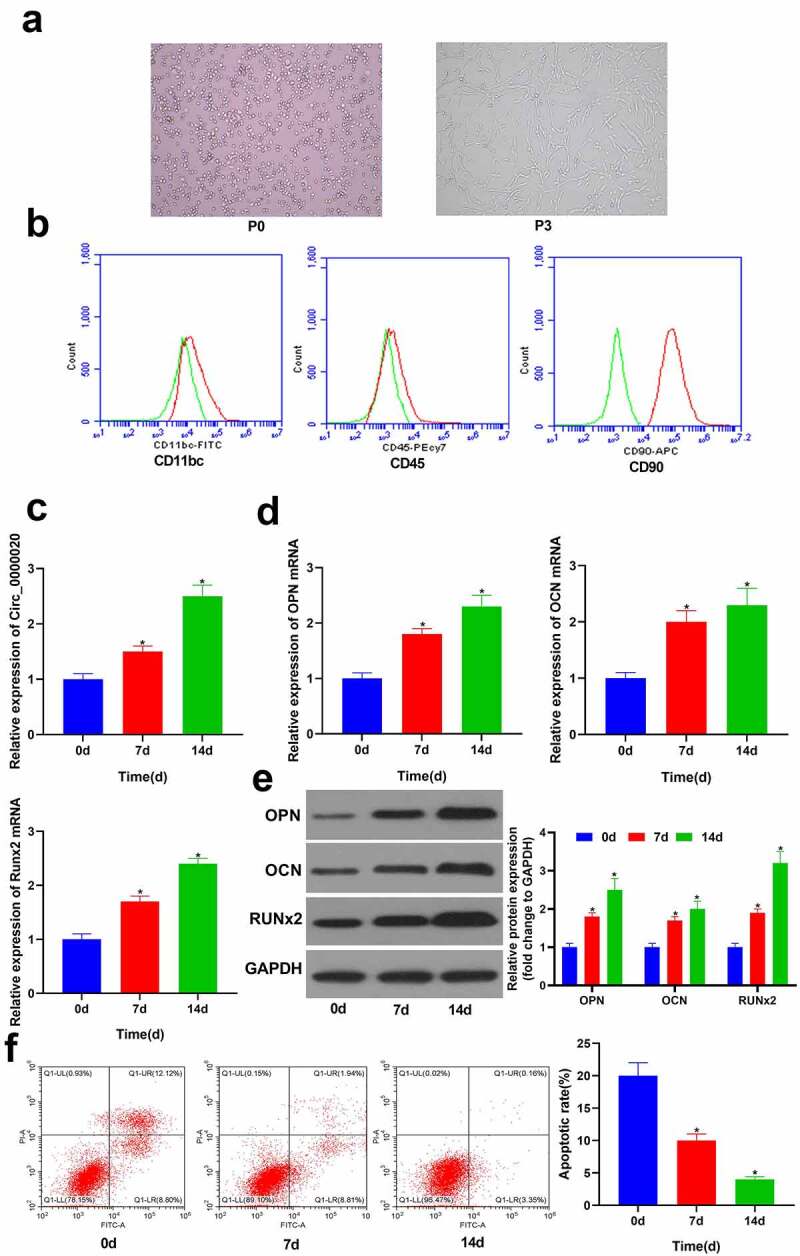
**Circ_0000020 expression amid BMSCs osteogenic differentiation**. (a) BMSCs were examined under a microscope. (b) Identifying BMSCs via flow cytometry. (c) Circ_0000020 expression after treating with osteogenic differentiation medium for 0, 7, and 14 days. (d) qRT-PCR detected osteogenic markers OPN, OCN and Runx2 mRNA expressions in BMSCs treated with osteogenic differentiation medium for 0, 7, and 14 days. (e) Western blot detected the of osteogenic markers OPN, OCN and Runx2 expressions in BMSCs treated with osteogenic differentiation medium for 0, 7, and 14 days. (f) Flow cytometry detected BMSCs apoptosis during osteogenic differentiation. *P < 0.05, compared with time = 0d

Circ_0000020 play important role in several diseases. However, its role is not well understood in OP and osteogenic differentiation. Therefore, we designed this study to evaluate the expression and functions of Circ_0000020 during BMSCs osteogenic differentiation. Moreover, we also studied the effects of Circ_0000020 interaction with miRNA miR-142-5p on its direct target gene BMP2. First of all, the primary BMSCs (P0) were isolated from SD rats, and cultured in media. Most of the cells were observed as round in shape (Figure 1a). After 3 hours of culture, few BMSCs began to grow statically and adhered to the wall. After 12 hours, adherent cells were observed in the plate. Cell colonies began to appear on 6th day, comprising of BMSCs clusters arranged radially. When the cells reached 80% confluency, BMSCs were harvested for further passages. After first passage, BMSCs had strong proliferation ability, and mostly were spindle-shaped. The third-generation BMSCs (P3) were fibroblast-like cells having flat or spindle-shaped bodies (Figure 1a). The cell surface markers of BMSCs were determined via flow cytometry. The findings revealed that CD90 was expressed in almost all BMSCs (P3), but hematopoietic cell antigen CD45 and neutrophil antigen CD11bc were not detected in BMSCs (P3). These findings confirmed that the cells isolated from SD rats were BMSCs (Figure 1b). Further, RT-qPCR was used to detect Circ_0000020 expression in BMSCs at 0 d, 7 d and 14 d of osteogenic differentiation. The findings revealed that with prolongated osteogenic differentiation, Circ_0000020 expression gradually increased (Figure 1c). Moreover, expression of osteogenic differentiation-related gene, that is, RUNX2, OPN, and OCN also gradually increased at protein and mRNA level with the prolonged osteogenic differentiation (Figure 1d-e). Flow cytometry findings revealed that with the increase in the osteogenic differentiation of BMSCs, there was significant decrease in the BMSCs apoptosis at 7 d and 14 d (figure 1f). These findings suggest that Circ_0000020 may have a regulatory impact on osteogenic differentiation.

### 3.2 Silencing Circ_0000020 inhibited BMSCs osteogenic differentiation

**Figure 2.**
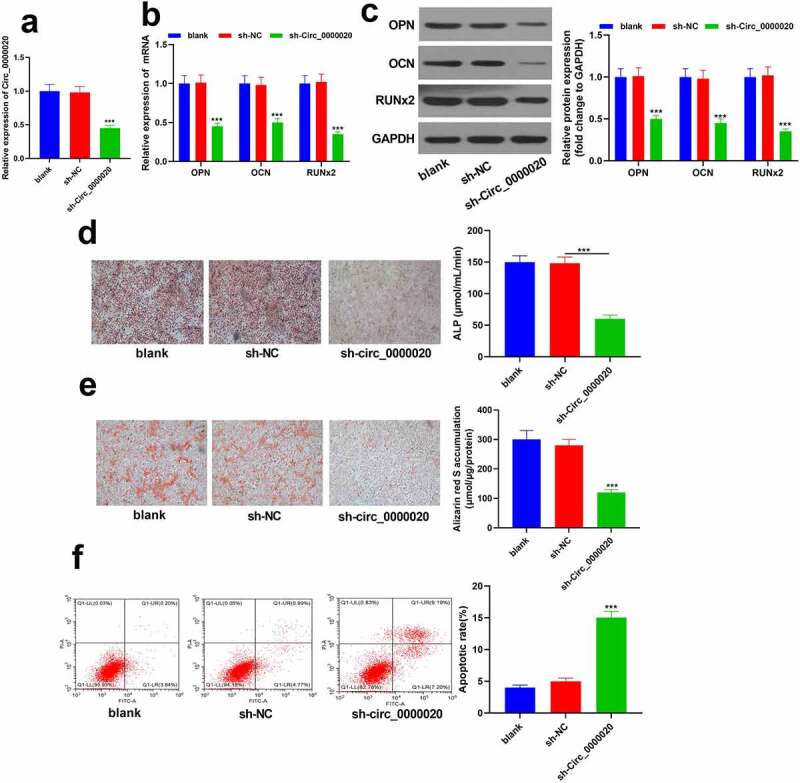
**Silencing Circ_0000020 inhibited BMSCs osteogenic differentiation**. (a) After transfecting sh Circ_0000020, qRT-PCR detected Circ_0000020 expression. (b) qRT-PCR detected osteogenic marker expressions, like OPN, OCN and Runx2 in BMSCs under the role of sh Circ_0000020. (c) Bone formation-related protein expressions (OPN, OCN and Runx2) in BMSCs after transfecting sh Circ_0000020. (d) ALP activity in BMSCs was detected on the 14th day after sh-Circ_0000020 treatment. (e) BMSCs treated with sh-Circ_0000020 were stained with alizarin red S on 14th day to show BMSCs mineralization ability. (f) Flow cytometry detected BMSCs apoptosis level after silencing Circ_0000020. **P < 0.01, ***P < 0.001, compared with sh-NC group

To determine the function of Circ_0000020 in osteogenic differentiation, Circ_0000020 gene-knockout BMSCs were established. RT-qPCR confirmed that Circ_0000020 expression in sh-Circ_0000020 group was significantly reduced (Figure 2a). Moreover, sh-Circ_0000020 treatment significantly reduced the expression of osteogenic markers (OPN, OCN and RUNX2) at mRNA and protein levels (Figure 2b-c). Meanwhile, ALP staining revealed that ALP content was significantly decreased after silencing of Circ_0000020 during BMSCs osteogenic differentiation (Figure 2d). Additionally, alizarin red S staining showed that silencing Circ_0000020 significantly inhibited BMSCs mineralization ability (Figure 2e). Flow cytometry analysis revealed that silencing of Circ_0000020 enhanced BMSCs apoptosis level as compared to the NC (figure 2f). These findings indicate that Circ_0000020 silencing inhibits BMSCs osteogenic differentiation.

### 3.3 Circ_0000020 acted as the ceRNA of miR-142-5p to positively regulate BMP2 level

**Figure 3.**
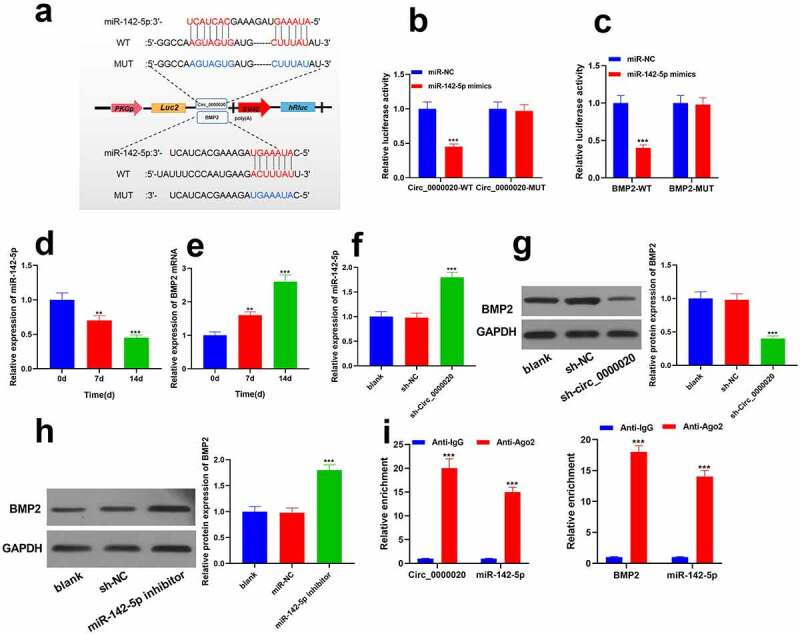
**Circ_0000020 directly as a sponge inserted the miR-142-5p of BMP2 gene transcript**. (a) Predicting the binding sites of miingR-142-5p with Circ_0000020 and BMP2 through bioinformatics analysis. (b-c) Circ_0000020-WT and Circ_0000020-mut luciferase activity in HEK293T cells treated with miR-142-5p mimic or NC. ***P < 0.001, compared with miR-NC. (d-e) miR-142-5p and BMP2 mRNA expressions in osteogenic differentiation medium at 0, 7, and 14 days. **P < 0.01, ***P < 0.001, compared with time = 0d. (f-g) QRT-PCR detected miR-142-5p and BMP2 expression after knocking down Circ_0000020. ***P < 0.001, compared with sh-NC. (h) QRT-PCR detected BMP2 level after transfecting miR-142-5p inhibitor. ***P < 0.001, compared with miR-NC. (i) RNA immunoprecipitation detected the binding of miR-142-5p to Circ_0000020 and BMP2. ***P < 0.001, compared with Anti-lgG

To further study the mechanism of Circ_0000020 regulating osteogenic differentiation, our work applied Starbase (http://www.sysu.edu.cn/403.html) and TargetScan (http://www.targetscan.org/vert_72/) to predict the Circ_0000020 interacting miRNAs. We found that Circ_0000020 has binding sites of miR-142-5p, whereas miR-142-5p targets the 3ʹUTR of BMP2 which is in important regulator in osteogenic differentiation (Figure 3a). Furthermore, dual luciferase reporter gene assay validated that relative luciferase activity was significantly reduced after the co-transfection of miR-142-5p and wild-type Circ_0000020 (Figure 3b). Moreover, co-transfection of miR-142-5p and wild-type BMP2 also reduced the luciferase activity (Figure 3c). However, miR-142-5p had no impact on mutant Circ_0000020 and mutant BMP2 (Figure 3b-c). Furthermore, RT-qPCR analysis determined the miR-142-5p and BMP2 expressions at 0 d, 7 d and 14 d of osteogenic differentiation. The findings revealed that miR-142-5p expression was significantly decreased in BMSCs osteogenic differentiation, whereas BMP2 mRNA expression was up-regulated (Figure 3d-e). Circ_0000020 silencing significantly increased miR-142-5p expression (figure 3f), while decreased BMP2 protein level (Figure 3g). Similarly, after inhibition of miR-142-5p, BMP2 protein level was considerably up-regulated (Figure 3h). RNA immunoprecipitation assay showed that the relative abundance of Circ_0000020 and BMP2 in anti-Ago2 group was higher than control group (Figure 3i). The above findings indicate that Circ_0000020 directly binds to miR-142-5p, thereby regulating BMP2 expression in BMSCs. Therefore, Circ_0000020 regulated BMP2 expression to induce osteogenic differentiation through miR-142-5p.

### 3.4 Circ_0000020 regulated BMSCs osteogenic differentiation through miR-142-5p/BMP2 pathway

**Figure 4.**
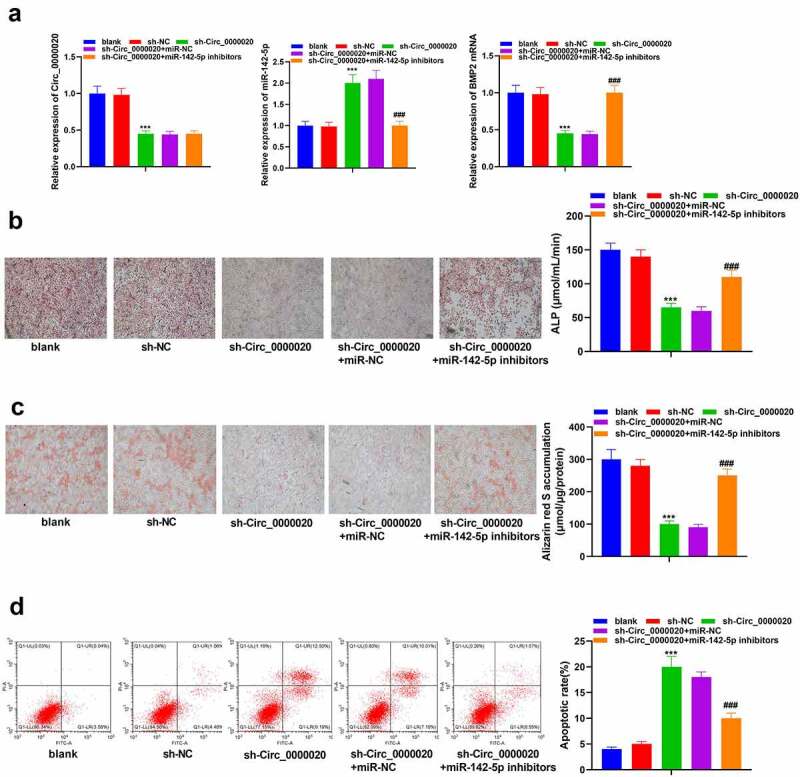
**MiR-142-5p inhibition reversed the role of Circ_0000020 gene knockout and inducing BMSCs osteogenic differentiation. After knocking out Circ_0000020 gene and transfecting miR-142-5p inhibitor**, (a) QRT-PCR detected Circ_0000020, miR-142-5p and BMP2 mRNA levels in BMSCs. (b) Detecting ALP activity in BMSCs. (c) Alizarin red S staining detected BMSCs mineralization ability. (d) Flow cytometry detected apoptosis level. ***P < 0.001, compared with sh-NC. ^###^*P* < 0.001, compared with sh Circ_0000020+ miR-NC

To further verify the interaction of miR-142-5p and Circ_0000020, we co-transfected miR-142-5p inhibitors and sh-Circ_0000020, and determined the regulatory role of Circ_0000020/miR-142-5p/BMP2 in osteogenic differentiation. Our results indicated that silencing of Circ_0000020 gene significantly reduced Circ_0000020 expression. After co-transfecting of sh-Circ_0000020 with miR-142-5p inhibitor, BMP2 mRNA expression was significantly up-regulated, whereas miR-142-5p expression was decreased (Figure 4a). Additionally, miR-142-5p inhibitors also reversed sh-Circ_0000020-induced changes in ALP and alizarin red S accumulation (Figure 4b-c). Moreover, the impact of sh-Circ_0000020 on promoting BMSCs apoptosis was attenuated by miR-142-5p inhibitors (Figure 4d). Based on these findings, we illustrated that inhibition of miR-142-5p rescues the sh-Circ_0000020 dependent decrease in osteogenic differentiation. Furthermore, Circ_0000020 regulated BMP2 expression via directly regulating miR-142-5p, thereby affecting BMSCs osteogenic differentiation.

### 3.5 Circ_0000020 regulated the BMP2/Smad pathway to induce osteogenic differentiation via regulating miR-142-5p

**Figure 5.**
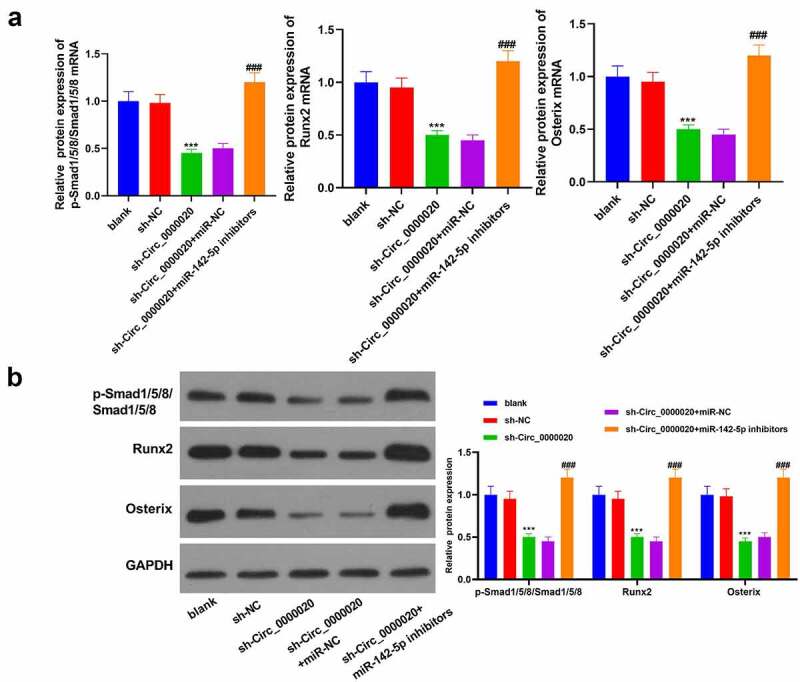
**Circ_0000020 regulated BMP2/Smad pathway via regulating miR-142-5p to induce osteogenic differentiation**. (a-b) QRT-PCR and western blot detected p-Smad1/5/8, Smad1/5/8, Runx2 and Osterix mRNA and protein expression levels in BMSCs. ***P < 0.001, compared with sh-NC. ^###^*P* < 0.001, compared with sh Circ_0000020+ miR-NC. (c) In general, Circ_0000020 induced osteogenic differentiation through down-regulating miR-142-5p and up-regulating BMP2

We have found that Circ_0000020 silencing up-regulates miR-142-5p expression, and down-regulates BMP2 expression. Moreover, BMP2 is the potential miR-142-5p target. Our study further probed into whether Circ_0000020 regulates BMP2 expression and its downstream signaling pathways through regulating miR-142-5p. We found that Circ_0000020 silencing considerably down-regulated p-Smad1/5/8, Runx2, Osterix mRNA and their protein levels (Figure 5a-b). Meanwhile, co-transfection of miR-142-5p inhibitors and sh-Circ_0000020 reversed BMP2, p-Smad1/5, Runx2 and Osterix protein levels which were induced by sh-Circ_0000020, suggesting that Circ_0000020 acts as a miR-142-5p sponge to regulate BMP2 expression and its downstream Smad signaling pathway. In general, Circ_0000020 induced osteogenic differentiation by up-regulating BMP2/SMAD pathway via down-regulation of miR-142-5p expression.

## 4 Discussion

Osteoporosis, a bone metabolism disorder, is resulting from imbalanced bone formation and resorption in body which may lead to the bone loss and some abnormal changes in the bone structure [27]. Presently, drugs like bisphosphonates and calcitonin are universally applied clinically to delay OP progression through suppression of osteoclast. However, it is difficult to improve trabecular bone microstructure in OP patients [28]. Therefore, exploring molecular mechanism regulating the osteogenic differentiation in BMSCs is very vital for treating OP. In this study, we identified a novel Circ_0000020 which has ability to promote ossification, and inhibit OP progression.

CircRNA may play important roles in regulating BMSCs osteogenic differentiation and bone formation [13]. So far, several CircRNAs have been reported to be associated with osteogenic differentiation. For instance, Circ_0024097 acts as ceRNA and sponge with miR-376b-3p which targets YAP1, and promotes osteogenic differentiation through the Wnt/β-catenin pathway [29]. Circ_0076690 is another well-documented ceRNA, proved to promote osteogenic differentiation via acting as miR-152 sponge [30]. Additionally, Circ_0076906 relieves osteoporosis and promotes osteogenic differentiation through miR-1305/OGN pathway [31]. But there are few reports about Circ_0000020, and it is very essential to understand the molecular mechanism of Circ_0000020 in osteogenic differentiation. This work confirmed that Circ_0000020 is up-regulated during BMSCs osteogenic differentiation. Therefore, we conducted further experiments to verify these results. Our further analysis showed that silencing of Circ_0000020 may inhibit osteogenic differentiation, which was confirmed by decreased expression of osteogenic marker (RUNX2, OPN, and OCN), ALP activity, and minerals accumulation, indicating that Circ_0000020 has a positive impact during BMSCs osteogenic differentiation. Therefore, we believed that Circ_0000020 is a CircRNA vital to the bone formation and osteogenic differentiation of BMSCs.

Additionally, since some CircRNAs have been reported as natural miRNA sponges [13] that interfere with downstream targets, our work also probed into whether Circ_0000020 regulates osteogenic differentiation through sponge binding to specific miRNAs. MiRNAs have also been reported to act pivotally in regulating osteogenic differentiation [32]. For example, miR-7b-5p prevents inhibiting fat differentiation via targeting IRS2 to promote BMSCs osteogenic differentiation and reduce OP [33]. MiRNA-27a-3p has been revealed to promote hMSC osteogenic differentiation via targeting ATF3 [34]. Additionally, miR-765, a well-proven miRNA, is available to target BMP6 via regulating BMP6/Smad1/5/9 signaling pathway, thereby inhibiting hMSC osteogenic differentiation [35]. Since miR-142-5p is predicted to bind Circ_0000020, our research focused on the miR-142-5p expression during BMSCs osteogenic differentiation and its relationship with Circ_0000020. In our research, miR-142-5p gradually declined after stimulating osteogenic differentiation. Additionally, after silencing Circ_0000020 gene, miR-142-5p expression in BMSCs was significantly up-regulated. Additionally, luciferase analysis also found that Circ_0000020 directly bound to miR-142-5p. Aiming to further study the role of miR-142-5p in BMSCs osteogenic differentiation, our work utilized miR-142-5p inhibitors to downregulate miR-142-5p expression, finding that inhibiting miR-142-5p reverses sh-Circ_0000020-induced osteogenic differentiation Inhibition and down-regulated osteogenic markers (Runx2, OCN and OPN). These osteogenic markers (Runx2, OCN and OPN) have no target or binding site for direct interaction of miR-142-5p or Circ_0000020. Whereas manipulating the expression of miR-142-5p or Circ_0000020 altered the expression of osteogenic markers (Runx2, OCN and OPN). This indicated that miR-142-5p and Circ_0000020 indirectly regulate the expression of osteogenic markers (Runx2, OCN and OPN). Moreover, with the alteration in the expression of osteogenic markers (Runx2, OCN and OPN) and overall osteogenic differentiation of BMSCs, the apoptosis rate of BMSCs also alters in inverse trend [36]. These findings indicate that Circ_0000020 regulates BMSCs osteogenic differentiation through sponging miR-142-5p as CeRNA. This is the first report so far to reveal that Circ_0000020 regulates BMSCs osteogenic differentiation via directly targeting miR-142-5p.

Based on reports, miR-142-5p functions pivotally in vertebrate bone development [14,37,38]. MiR-142-5p is available to inhibit osteoblast function via targeting VCAM-1 [14]. But studies on miR-142-5p regulating osteogenic differentiation through BMP2/Smad pathway have not been reported yet. Our work found that BMP2 is the direct miR-142-5p target, and that Circ_0000020 regulates BMP2/Smad signaling pathway through sponging miR-142-5p. This is the first study to report that Circ_0000020/miR-142-5p/BMP2/SMAD axis regulates BMSCs osteogenic differentiation.

Although first is to reveal the new molecular mechanism of Circ_0000020/miR-142-5p/BMP2 axis which regulates BMSCs osteogenic differentiation, there is still much work needed to understand the role of Circ_0000020 during BMSCs osteogenic differentiation and OP pathogenesis. Since the OP situation in animal models is more complex as compared to cell models, more research is needed in animal bone models. Therefore, the related molecular mechanism of Circ_0000020/miR-142-5p/BMP2 axis needs further verification animal model, which will provide further evidence for the role of Circ_0000020/miR-142-5p/BMP2 axis in regulating BMSCs osteogenic differentiation. Moreover, it is also required to study the role of Circ_0000020 in OP animal model Circ_0000020 specific knock-out animal model.

## 5 Conclusion

Overall, our work proves that Circ_0000020 is upregulated during osteogenic differentiation of BMSCs. Moreover, Circ_0000020 regulate BMSCs osteogenic differentiation via sponging miR-142-5p as CeNA to and regulates the BMP2/Smad pathway. Our research is the first study to reveal the molecular regulation of osteogenic differentiation of BMSCs through Circ_0000020/miR-142-5p/BMP2 axis, thus provides a potential therapeutic target for OP.

## Highlights

1. Circ_0000020 expression is up-regulated during osteogenic differentiation of BMSCs.
2. Silencing of Circ_0000020 induced apoptosis and restrict osteogenic differentiation in BMSCs.
3. Circ_0000020 binds to the miR-142-5p, thereby increasing the expression of BMP2 in BMSCs.
